# EphrinB2 sharpens lateral motor column division in the developing spinal cord

**DOI:** 10.1186/s13064-015-0051-9

**Published:** 2015-10-26

**Authors:** Maëva Luxey, Julien Laussu, Alice Davy

**Affiliations:** Centre de Biologie du Développement, CNRS, 118 Route de Narbonne, 31062 Toulouse, France; Université de Toulouse, Toulouse, France; Institut de Recherche Clinique de Montréal, 110 avenue des Pins Ouest, Montréal (Québec), H2W 1R7 Canada

**Keywords:** Spinal cord, Motor neurons, Motor columns, Eph/ephrin, Cell sorting, Mouse

## Abstract

**Background:**

During sensori-motor circuit development, the somas of motoneurons (MN) are distributed in a topographic manner in the ventral horn of the neural tube. Indeed, their position within the lateral motor columns (LMC) correlates with axonal trajectories and identity of target limb muscles. The mechanisms by which this topographic distribution is established remains poorly understood. To address this issue, we assessed the role of ephrinB2 in MN topographic organization in the developing mouse spinal cord.

**Results:**

First, we used a reporter mouse line to establish the spatio-temporal expression pattern of *EfnB2* in the developing LMC*.* We show that early in LMC development, ephrinB2 is differentially expressed in MN of the lateral versus medial LMC, suggesting a possible role in MN sorting and/or migration. We demonstrate that while MN-specific excision of *EfnB2* did not perturb specification or migration of MN, conditional loss of ephrinB2 led to the blurring of the LMC divisional boundary and to errors in the selection of LMC axon trajectory in the limb.

**Conclusions:**

Altogether, our study uncovered a novel cell autonomous role for ephrinB2 in LMC MN thus emphasizing the prevalent role of this ephrin member in maintaining cell population boundaries.

**Electronic supplementary material:**

The online version of this article (doi:10.1186/s13064-015-0051-9) contains supplementary material, which is available to authorized users.

## Background

A recurring theme in the organization of the central nervous system is the grouping of neurons innervating the same target. Because neurons are often born at a distance from their final settling position, the establishment of this topography requires complex migration and clustering processes [1–3]. In the ventral spinal cord, motoneurons (MN) are grouped in motor columns according to their identity and to their target muscle. MN innervating the limb settle in the lateral motor column (LMC) which is further divided into two divisions: lateral LMC (LMCl) composed of MN innervating the dorsal part of the limb and medial LMC (LMCm) formed by MN innervating the ventral part of the limb. Both LMCl and LMCm occupy stereotypical positions within the LMC [4, 5].

Shortly after exiting the cell cycle at the basal side of the ventricular zone of the spinal cord, MN migrate radially toward the marginal zone. A second phase of tangential migration followed by coalescence of same-identity MN soma gives rise to the stereotypical organization of motor columns in the ventral horn of the spinal cord. The different motor columns are characterized by the expression of different sets of transcription factors. For instance, all somatic MN express HB9, whereas Foxp1 is expressed in all LMC MN at high level. Lastly, LMCl and LMCm MN express Lim1 (Lhx1) and Islet1 respectively [6]. All these transcription factors have been shown to contribute to the establishment of MN organization in columns. Indeed, gain and loss of function of Foxp1, Lim1, Islet1 and HB9 lead to important defects of MN positioning within the spinal cord along with a range of axon pathfinding defects [7–12]. Although the role of these transcription factors in specifying the identity and position of MN within the spinal cord is well established, little is known on their potential effector genes. A handful of molecular players involved in MN soma migration have been identified in the last few years, for instance, Reelin, an extracellular protein well known for its role in controlling radial migration of cortical neurons, was shown to control tangential migration of LMC MN [10]. Moreover, members of the cadherin family, especially type II cadherins, have also been involved in this migratory process [13] and a recent study identified axon guidance molecules of the Slit/Robo and Netrin/DCC pathways as repulsive and attractive cues, respectively, for MN cell bodies in the ventral spinal cord [14]. Of note, cadherins are the only molecular players identified to date in the control of MN soma clustering [15, 16].

The Eph/ephrin family has been widely involved in mechanisms of cell sorting, cell migration and axon guidance during development [17]. In the sensory-motor circuit innervating the limb, this family of proteins has been shown to control guidance of motor axons, sorting between motor and sensory axons and synaptogenesis [18, 19]. Amongst all members of the Eph/ephrin family, ephrin-B2 and EphA4 seem to play a particularly important role in cell sorting. For instance in the zebrafish hindbrain, the ephrinB2/EphA4 pair is responsible for the formation and maintenance of rhombomeres boundaries [20, 21] and this same pair was shown to be involved in maintaining the anteroposterior patterning of somites [22, 23]. Interestingly, rostrocaudal displacement of a MN pool innervating the hindlimb has been reported in EphA4 deficient mice [24] as well as LMC axon guidance defects [25]. Concerning ephrinB2, previous work has shown that it plays a dual role in controlling LMC MN axon guidance. As a ligand expressed in the limb mesenchyme, it activates Eph signaling in growing LMCm axons thus repelling them from the dorsal limb [26]. In addition, experiments in the chick showed that expression of ephrinB2 in LMCl attenuates Eph signaling in these axons thus allowing them to invade the dorsal limb [27].

Herein, we asked whether ephrinB2 regulates the migration, position and/or grouping of MN soma in the mouse spinal cord. Using the *EfnB2:H2BGFP* reporter mouse line, we established the spatial and temporal expression pattern of ephrinB2 in MN of the LMC. We show that ephrinB2 is differentially expressed in LMCl vs. LMCm MN at the time these two populations coalesce, with a higher expression in LMCl MN. We confirm that ephrinB2 cell autonomously controls the guidance of LMCl axons in the mouse and we provide evidence that conditional loss of *EfnB2* in MN impairs clustering of LMC MN soma without affecting their migration.

## Results and discussion

### Dynamic expression of ephrinB2 in the ventral spinal cord

**Fig. 1:**
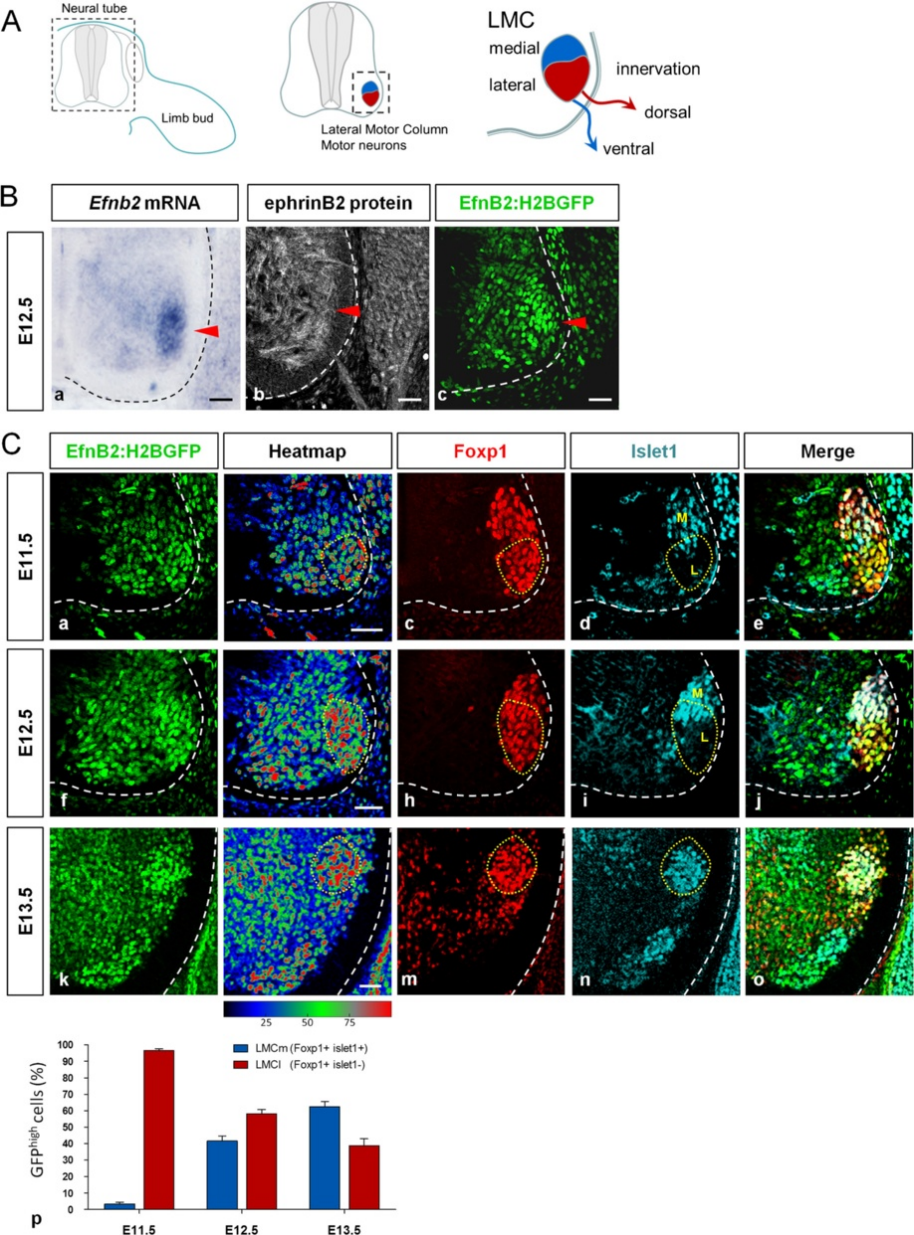
Expression pattern of ephrinB2 in the ventral neural tube. **a**/ Schematic representation of transverse sections of mouse embryo at brachial level, with a focus on the LMC in the ventral horn. **b**/ Detection of *EfnB2* mRNA expression (**a**), ephrinB2 protein expression (**b**) and H2BGFP expression driven from the endogenous *EfnB2* promoter (**c**) in the ventral neural tube of E12.5 embryos at brachial level. Arrowheads indicate the position of the LMC. C/ Transverse vibratome sections of E11.5 (a-e), E12.5 (f-j), E13.5 (k-o) *EfnB2* ^+/GFP^ embryos were immunostained for Foxp1 (c, h, m; red) and Islet1 (d, i, n; blue) labeling LMC and LMCm neurons respectively. Epifluorescence of GFP (a, f, k; green) is also represented on heat maps to visualize cells expressing high levels of ephrinB2 (GFP^high^ in red). Dotted lines mark Foxp1+/GFP^high^ cells. Proportion of Foxp1+/GFP^high^ MN expressing Islet1 (LMCm, blue) or not (LMCl, red) in E11.5, E12.5 and E13.5 embryos (*n* = 3 for each stage) (p). Error bars indicate s.e.m.; Scale bars: 50 μm

To investigate the potential role of ephrinB2 in MN of the LMC, we first analyzed its expression pattern in the ventral horn of the neural tube in mouse embryos. At E12.5, we observed a strong expression of *EfnB2* mRNA in the region corresponding to the area containing differentiated LMC neurons (Fig. 1Ba). Immunofluorescent staining of the endogenous ephrinB2 protein revealed a pattern consistent with an expression in the LMC, however, the diffuse quality of the staining due to the membrane-bound nature of the protein prevented further characterization of the expression pattern (Fig. 1Bb). To circumvent this limitation inherent to cell surface proteins, we used a reporter mouse line in which the endogenous *EfnB2* promoter drives expression of a nuclear Green Fluorescent protein (GFP) (*EfnB2:H2BGFP;* [23]. At E12.5, intense epifluorescence could be visualized in several cell populations in the ventral horn of the spinal cord (Fig. 1Bc). This mouse line has the great advantage of allowing co-labeling between nuclear GFP and transcription factors which are classically used as identity markers of the different types of MN in the LMC. The transcription factor Foxp1 is commonly used as a marker of LMC neurons while lateral and medial subdivisions are typically identified by the expression of the transcription factors Lim1 or Islet1, respectively. Alternatively, the identity of the lateral LMC motor neurons can be determined based on the presence of Foxp1 and absence of Islet1 [9]. Thus, for most of our analyses, we used the combination of transcription factors Foxp1^+^/Islet1^+^ to mark MN of the LMCm and Foxp1^+^/Islet1^−^ to label those of the LMCl. To establish the spatio-temporal expression pattern of ephrinB2 in LMC neurons, we monitored the expression of H2BGFP in the spinal cord between E11.5 and E13.5 at brachial level, a developmental period that encompasses coalescence and tangential migration of LMC MNs. At all developmental stages analyzed, we observed expression of H2BGFP in a large number of nuclei but at variable levels. To highlight cells with the strongest expression of ephrinB2 (GFP^high^), we used heat maps visualization of GFP fluorescence on transverse sections. Co-staining with Foxp1 and Islet1 indicated that at E11.5, the majority of Foxp1^+^/GFP^high^ nuclei were Islet1^−^ suggesting that the majority of cells expressing high levels of ephrinB2 are LMCl neurons (Fig. 1c). To confirm that GFP^high^ nuclei were LMCl MN, we performed Lim1 immunostaining. As expected, at E11.5, the majority of GFP^high^ nuclei were also Lim1^+^ (Additional file 1: Figure S1A). From E12.5 to E13, an increasing proportion of Islet1^+^ LMC MN expressed strongly the GFP indicating that high expression of ephrinB2 is no longer restricted to one division (Fig. 1c). Altogether, these results demonstrate that ephrinB2 is differentially expressed in the two divisions of the LMC, in a transient manner, at the time of MN soma grouping. Several Eph receptors are expressed in LMC MN (Additional file 1: Figure S2) [9, 26]. Amongst these, EphB1 and EphA4 have been shown to be differentially expressed in LMCl vs. LMCm MN and to play a role in controlling dorsal vs. ventral innervation of the limb by these axons [9, 26]. It thus would be interesting to test whether similar to ephrinB2, these receptors may also control segregation of LMC MN soma. After E11.5, ephrinB2 expression is no longer restricted to one division indicating that expression of ephrinB2 becomes independent of MN divisional identity. At these later stages LMC divisions are further divided into MN pools which contain neurons innervating specific muscles targets [3]. It is thus tempting to speculate that differential expression of ephrinB2 may correlate with MN pool identity.

### EphrinB2 is required for guidance of LMCl axons

**Fig. 2:**
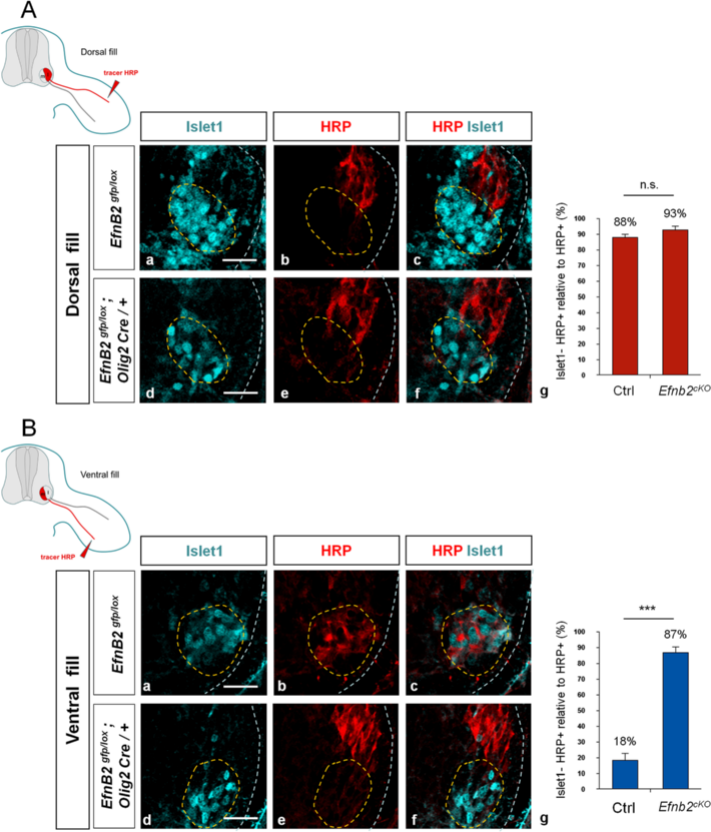
cKO embryos exhibit axon guidance defects. **a**/ Transverse vibratome sections of E12.5 control (*EfnB2* ^lox/GFP^) (a-c) and *Efnb2* ^*cKO*^ (*EfnB2* ^lox/GFP^; *Olig2-Cre*) embryos (d-f) injected with HRP into dorsal forelimb shank muscles and stained for HRP (b, e) and Islet 1 (a, d). Dotted lines indicate LMCm MN. Proportion of HRP+/Islet 1- neurons (g) in control (*n* = 3) and *Efnb2* ^*cKO*^ (*n* = 3) embryos. **b**/ Transverse vibratome sections of E12.5 control (*EfnB2* ^lox/GFP^) (a-c) and *Efnb2* ^*cKO*^ (*EfnB2* ^lox/GFP^; *Olig2-Cre*) embryos (d-f) injected with HRP into ventral forelimb shank muscles and stained for HRP (b, e) and Islet 1 (a, d). Dotted lines indicate LMCm MN. Proportions of HRP+/Islet 1- neurons (g) in control (*n* = 3) and *Efnb2* ^*cKO*^ embryos (*n* = 3). Error bars indicate s.e.m.; ***P < 0.001; ns = non significant (unpaired two-sample *t*-tests). Scale bars: 50 μm

To address the cell autonomous function of ephrinB2 in LMC MN, we generated embryos harboring a conditional loss of *EfnB2* in MN (*EfnB2*^*cKO*^). *EfnB2* was selectively excised from MN using the *Olig2-Cre* allele [28] with *EfnB2*^*cKO*^ embryos carrying one conditional allele and one null allele of *EfnB2* (*EfnB2*^*lox/H2BGFP*^*; Olig2Cre*). We validated the loss of *EfnB2* expression in MN of mutant embryos by in situ hybridization (Additional file 1: Figure S3A). Loss of *EfnB2* did not affect the specification of LMC MN (Additional file 1: Figure S3B) but we observed a reduction in the number of MN (unpublished observation). EphrinB2 has been shown previously to play a cell autonomous role in LMC MN axon pathfinding in the chick embryo [27]. To test whether this function is conserved in the mouse, we performed retrograde labeling of MN from the forelimb. We injected the HRP tracer into the dorsal or ventral limb musculature of E12.5 embryos and assessed the identity of the retrogradely labeled neurons. In control embryos, as expected, the majority of neurons labeled following dorsal fill belonged to the LMCl (Foxp1^+^ Islet1^−^). Similar results were observed in *EfnB2*^*cKO*^ embryos indicating that LMCl project normally to the dorsal limb in absence of ephrinB2 (Fig. 2a). In contrast, *EfnB2*^*cKO*^ embryos exhibited aberrant ventral projections from the LMCl since 87 % of ventrally filled neurons were Islet1^−^ in absence of ephrinB2, compared to 19 % in control embryos (Fig. 2b). Thus, in absence of ephrin-B2 expression, a fraction LMCl MN redirected their axons from dorsal to ventral limb mesenchyme. Unexpectedly, in *EfnB2*^*cKO*^ embryo, only 13 % of axons innervating the ventral limb bud are LMCm motor axons (Islet1^+^). One possible explanation for this low number could be that in absence of ephrinB2 a significant fraction of LMCm axons is redirected more ventrally towards body wall muscles. Innervation of body wall muscles has been described as a ternary trajectory choice for a subset of LMC axons in wild type conditions [29]. Further, redirection of axonal projections due to elevated occupancy of normal targets has been reported in other contexts [30]. These data indicate that similar to the chick, ephrin-B2 is required to control pathfinding of LMC axons in a cell autonomous manner in the mouse.

### EphrinB2 does not control LMC soma migration

**Fig. 3:**
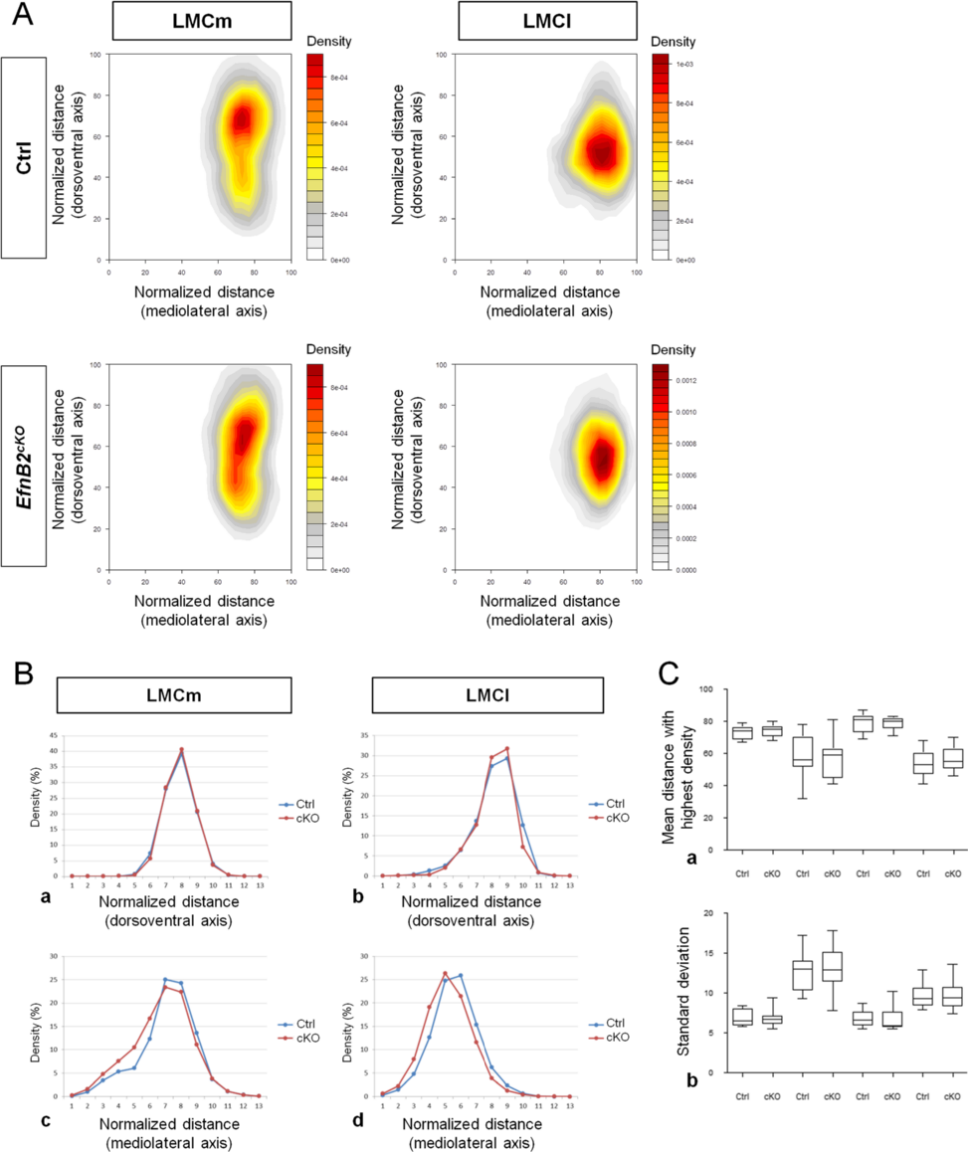
EphrinB2 does not control migration of MN. A/ Heat map representation of the position, in the dorsoventral and mediolateral axes, of LMCm and LMCl neurons in the ventral horn of the spinal cord, from control (*n* = 13) and *Efnb2* ^*cKO*^ (*n* = 11) E13 embryos. B/ Nuclei density of LMCm (a, c) and LMCl (b, d) plotted against distance from the reference point in the dorsoventral (a, b) and mediolateral (c, d) axes, in control (blue lines) and *Efnb2* ^*cKO*^ (red lines) embryos. **c/** Mean distance with the highest nuclei density for LMCm and LMCl neurons (**a**) Standard deviation of distances with highest nuclei density (**b**) Error bars indicate s.e.m.; ns = non significant (unpaired two-sample *t*-tests).

It has been shown that Reelin is required for the tangential migration and proper positioning of LMCl neurons within the ventrolateral spinal cord [10]. Indeed, VLDLR, a Reelin receptor, exhibits a restricted expression in LMCl neurons and one effector of Reelin signaling, Dab1, colocalizes with VLDLR in LMCl neurons. Yet, Dab1 mutant embryos show a defect in the positioning of LMC MN more important than Reelin mutants suggesting that another signaling pathway using the effector Dab1 could be involved in parallel. A recent study on the developing cortex revealed that ephrinBs are able to form a complex with VLDLR and are able to activate Dab1 thus modulating the migration of cortical neurons [31], raising the possibility that ephrinB2 might play a cell autonomous role in controlling migration of LMC MN. To test this hypothesis, we recorded the position of LMCm (Foxp1^+^/Islet1^+^) and LMCl (Foxp1^+^/Islet1^−^) nuclei on sections from control and *EfnB2*^*cKO*^ embryos. The analysis was performed at E13, a stage at which the majority of LMC neurons has completed its tangential migration. Positions were recorded by measuring the distance of each nucleus to a reference point (see Methods section), both in the mediolateral and dorsoventral axes. In order to normalize natural variations in the size of the spinal cord between embryos, MN position was defined as a percentage of the maximum width of the spinal cord. These normalized values were plotted on a 2D-map of the ventral horn of the neural tube with the X axis representing the mediolateral position and Y axis representing the dorsoventral position of each nucleus (Fig. 3a). No gross mis-positioning of LMCl and LMCm within the spinal cord could be observed in absence of ephrinB2. To more precisely compare positions of LMC MN soma in the two genotypes, nuclei density was plotted according to their distribution in each axis (Fig. 3b left). Also, no difference in MN distribution was observed between control and *EfnB2*^*cKO*^ embryos. This was confirmed by performing a statistical analysis on the mean distance with highest nuclei density for each division and on each axis (Fig. 3b right). Altogether, this detailed analysis suggests that ephrinB2 is not required to control radial or tangential migration of LMC MN soma within the spinal cord.

### EphrinB2 is required to maintain sharp segregation between LMC MN

**Fig. 4:**
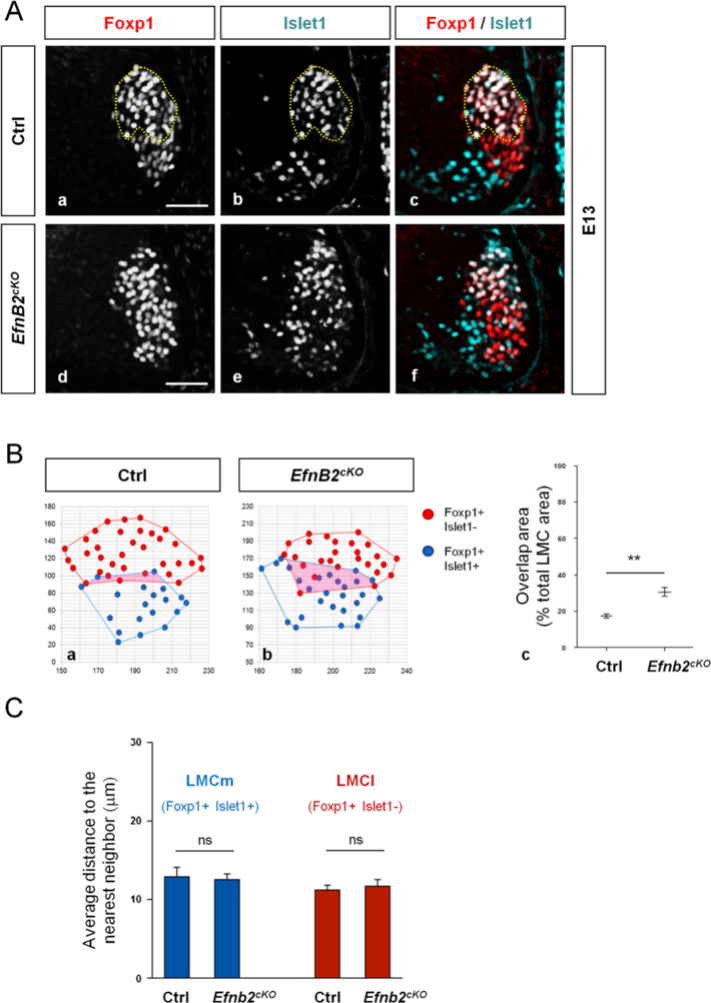
EphrinB2 controls grouping of MN into LMC divisions. A/ Transverse vibratome sections of E13 control (**a**) and *EfnB2* ^cKO^ (**b**) embryos were immunostained to detect Foxp1 (red) and Islet1 (blue). B/ Schematic representation of the image analyses used for quantification (**a**, **b**). Quantification of the surface of overlap in images from control and *EfnB2* ^cKO^ embryos (*n* = 4 embryos per genotype) C/ Quantification of the distance to the nearest neighbor of the same identity in LMCm and LMCl of control and *EfnB2* ^cKO^ embryos (*n* = 4 embryos per genotype). Error bars indicate s.e.m.; **P < 0.01; ns = non significant (unpaired two-sample *t*-tests). Scale bars: 50 μm

Although ephrinB2 does not control the general ventrolateral location of the LMC, we next examined whether loss of ephrinB2 influenced the grouping of MNs in the two subdivisions of the LMC. On sections from *EfnB2*^*cKO*^ embryos immunostained for Foxp1 and Islet1, we occasionally observed LMCm neurons positioned within the LMCl (Fig. 4a). To quantify this phenotype, we measured surfaces encompassing all LMCm (Foxp1+ Islet1+) and all LMCl (Foxp1+ Islet1-) neurons on sections from control and *EfnB2*^*cKO*^ embryos. From these measurements, we deduced the area of overlap between LMCl and LMCm, reasoning that a sharp boundary between subdivisions would produce a small overlap while mispositioning of nuclei within divisions would produce a large overlap. In *EfnB2*^*cKO*^ the surface of overlap between LMCl and LMCm was increased compared to control embryos (Fig. 4b) indicating that ephrinB2 plays a role in maintaining proper grouping of MN in each LMC subdivisions. To assess whether this phenotype was due to increased mixing or dispersion of LMCl and LMCm nuclei, we measured the average distance between nuclei within each subdivisions. There was no change in the distance between nuclei in absence of ephrinB2 (Fig. 4c), indicating that the increased surface of overlap is not due to increased dispersion between nuclei. Together, these results suggest that differential expression of ephrinB2 in LMC MN is required to maintain segregation of MN in each LMC divisions and prevent mixing of LMCl and LMCm MN soma. Although statistically significant, the disorganization of LMC MN grouping observed in *EfnB2* cKO was modest. Several members of the Eph receptor and ephrin families are expressed in LMC MN [18], raising the possibility that these members play partly redundant functions. In this context, it would be interesting to investigate LMC MN topography in double or triple Eph/ephrin mutant embryos. Little is known on proteins regulating clustering of MN. Similar to MN identity which is regulated by a combination of transcription factors, it has been proposed that coalescence of MN into divisions and pools may be regulated by combinatorial sets of adhesion molecules [32]. Thus far only cadherins have been proposed to control in this process [13, 16, 33]. Given the prevalent role of ephrinB2 in maintaining cell population boundaries in various developmental contexts our results suggest that ephrinB2 is a novel actor participating in this combinatorial code of adhesion/repulsion molecules. However, based on our data we cannot completely exclude that the observed phenotype results from subtle alterations of migration.

## Conclusions

Ordered topography of neuronal soma requires the delicate orchestration of various processes including neuronal specification, radial and tangential migration as well as soma coalescence. While mechanisms controlling specification of LMC MN are fairly well characterized, the actual molecular effectors –cytoplasmic factors, cytoskeleton proteins and cell surface receptors- involved in MN migration and grouping remain elusive. We show here that in addition to its well characterized role in guiding motor axons [18], Eph:ephrin signalling plays a role in setting up the topography of MN soma, lending support to the notion that identical factors control several steps of myotopic organisation.

## Methods

### Animals

*Efnb2*^*+/GFP*^, *Efnb2*^*lox/lox*^ and *Olig2-Cre* mice were as described [23, 28, 34]. *Efnb2*^*cKO*^ (*EfnB2*^*lox/H2BGFP*^; *Olig2-Cre*) and control embryos were collected from the same litters. Control genotypes included *Efnb2*^*lox/H2BGFP*^, *Efnb2*^*+/H2BGFP*^, *Efnb2*^*+/H2BGFP*^; *Olig2-Cre*. All animal procedures were pre-approved by the “Comité d’éthique Régional” (protocol number: MP/07/21/04/11).

### Genotyping

REDExtract-N-Amp™ Tissue PCR kit was used for all genotyping PCR. Yolk sac was used to genotype embryos while tail tissue was used to genotype pups. The following primers were used for the *EfnB2* floxed allele: Cs1 5′-CTTCAGCAATATACACAGGATG-3′and Cas1 5′-TGCTTGATTGATTGAAACGAAGCCCGA-3′; for the Cre allele: Cre-S 5′- ACGGAAATCCATCGCTCGACCA-3′ and Cre-AS 5′-GTCCGGGCTGCCACGACCAA-3′. H2BGFP was detected by epifluorescence on whole embryos.

### In situ hybridization

ISH was performed on transversal thick vibratome sections at brachial level of E12.5 embryos. Briefly, embryos were fixed in 4 % paraformaldehyde (PFA) and dehydrated in ethanol. Following rehydration, embryos were sectioned and 70 μm sections at brachial level were treated with proteinase K (10 μg/ml in PBS/0.1 % Tween-20) for 7 min at room temperature and subsequently post-fixed in PFA/glutaraldehyde solution. Embryos were incubated overnight at 65 °C in hybridization buffer (50 % formamide, 5x SSC (pH 6), 0.1 % SDS, 50 μg/ml heparin, 500 μg/ml yeast RNA) containing the labelled probe. Embryos were washed twice with solution I (5x SSC, 50 % formamide, 0.1 % SDS) at 65 °C and 3 times in solution III (2x SSC, 50 % formamide, 0.1 % SDS) at 65 °C, rinsed in TBS/0.1 % Tween-20 and incubated overnight in blocking buffer (TBS with 2 % goat serum, 0.1 % blocking reagent (Roche), 0.1 % Tween-20) containing an AP-labelled anti-DIG antibody (1/2000) (Roche). NBT/BCIP was used as a substrate for the Alkaline Phosphatase.

### Immunostaining

Embryos were fixed overnight at 4 °C in 4 % PFA (or Ethanol/Acetic acid for ephrinB2 staining). Thick vibratome sections (70 μm) at brachial level were collected in PBS, washed in PBS/ Triton 0.5 % and blocked in PBS containing 1 % BSA/0.1 % Triton. Sections were incubated with primary antibodies against Foxp1 (gift from Dr. Novitch: 1/2000), Islet1 (gift from Dr. Jessell: 1/1000), Islet1/2 (Hybridoma Bank: 1/100), Lim1 (gift from Dr. Jessell: 1/1000), HRP (Jackon Immuno Research:1/2000) or ephrinB2 (R&D systems: 1/50) overnight at 4 °C. The ephrinB2 antibody was validated previously by us and others [35–38]. Secondary antibodies were applied for 1 h at room temperature. Confocal microscopy was carried out on a Leica SP5 confocal.

### HRP retrograde labeling of motor neurons

Retrograde labeling of mouse motor neurons using HRP (Roche) as tracer was performed as described [39]. HRP was injected into either dorsal or ventral hindlimb shank musculature of E12.5 mouse embryos. LMC MN nuclei were visualized using H2BGFP expressed from the *EfnB2* promoter (not shown in the figure). MN were considered HRP positive when the nucleus was surrounded by HRP labelling.

### Image analyses

Heatmap representation was obtained using the MatLab software. For each gray value in an 8-bit image, pixels were counted in the defined interval and then transformed in a heatmap with calibration bar representing the relative abundance of GFP (0 to 100 %). GFP^high^ cells are cells with a 100 % relative abundance of GFP (red cells on heatmaps). Calculation of the distance to the nearest neighbor was done using the software R (see Additional file 2 for details). The measurement of surface overlap was done as described in (Laussu et al. Antagonistic Eph:ephrin signaling patterns the ventral neural tube, Submitted).

## Additional files

Additional file 1:
**Figure S1.** Co-expression of ephrinB2 and Lim1. Transverse vibratome sections of E11.5 (a-e), E12.5 (f-j), E13.5 (k-o) *EfnB2*+/GFP embryos were immunostained for Lim1 (c, h, m; red), Foxp1 (d, i, n; blue) showing LMCl and LMC neurons respectively. Epifluorescence from the GFP (a, f, k; green) is also represented on heat maps (b, g, l) to visualize cells expressing high levels of ephrinB2 (GFPhigh in red). Dotted lines mark Foxp1+/GFPhigh cells. Scale bars: 50 μm. **Figure S2.** Eph receptors are expressed in LMC MN. Expression of *EphA4* (a), *EphB2* (b) and *EphB3* (c) was detected on transverse vibratome sections of E12.5 embryos by in situ hybridization. Scale bars: 50 μm. **Figure S3.** Conditional excision of *Efnb2* in MN does not affect specification of LMC MN. A. Transverse vibratome sections of control (*EfnB2*+/GFP) and *Efnb2cKO* (*EfnB2*lox/GFP*; Olig2-Cre*) E13.5 embryos were processed for *Efnb2* in situ hybridization. Arrowheads indicate the position of the LMC. B. Transverse vibratome sections of control (*EfnB2*+/GFP) and *Efnb2cKO* (*EfnB2*lox/GFP*; Olig2-Cre*) E13 embryos were immunostained for Foxp1 and Islet1 (see Fig. 4). The graph shows the proportion of LMCm and LMCl MN in both genotypes. Error bars indicate s.e.m.; ns = non significant. Scale bars: 200 μm. (PDF 259 kb) Additional file 2:
**Nearest Neighbor algorithm for R software.** (DOC 28 kb)

## Abbreviations

GFP: Green fluorescent protein
LMC: Lateral motor column
LMCl: Lateral lateral motor column
LMCm: Medial lateral motor column
MN: Motor neurons
