# Supplementary material for: EphrinB2 sharpens lateral motor column division in the developing spinal cord
Source: Neural Dev. 2015 Oct 26;10:25. doi: 10.1186/s13064-015-0051-9 (PMC4624581; doi:10.1186/s13064-015-0051-9)
Supplement: Additional file 1: — Figure S1. Co-expression of ephrinB2 and Lim1. Transverse vibratome sections of E11.5 (a-e), E12.5 (f-j), E13.5 (k-o) EfnB2+/GFP embryos were immunostained for Lim1 (c, h, m; red), Foxp1 (d, i, n; blue) showing LMCl and LMC neurons respectively. Epifluorescence from the GFP (a, f, k; green) is also represented on heat maps (b, g, l) to visualize cells expressing high levels of ephrinB2 (GFPhigh in red). Dotted lines mark Foxp1+/GFPhigh cells. Scale bars: 50 μm. Figure S2. Eph receptors are expressed in LMC MN. Expression of EphA4 (a), EphB2 (b) and EphB3 (c) was detected on transverse vibratome sections of E12.5 embryos by in situ hybridization. Scale bars: 50 μm. Figure S3. Conditional excision of Efnb2 in MN does not affect specification of LMC MN. A. Transverse vibratome sections of control (EfnB2+/GFP) and Efnb2cKO (EfnB2lox/GFP; Olig2-Cre) E13.5 embryos were processed for Efnb2 in situ hybridization. Arrowheads indicate the position of the LMC. B. Transverse vibratome sections of control (EfnB2+/GFP) and Efnb2cKO (EfnB2lox/GFP; Olig2-Cre) E13 embryos were immunostained for Foxp1 and Islet1 (see Fig. 4). The graph shows the proportion of LMCm and LMCl MN in both genotypes. Error bars indicate s.e.m.; ns = non significant. Scale bars: 200 μm. (PDF 259 kb) [file 13064_2015_51_MOESM1_ESM.pdf]

Sup Figure 1, Luxey et al.

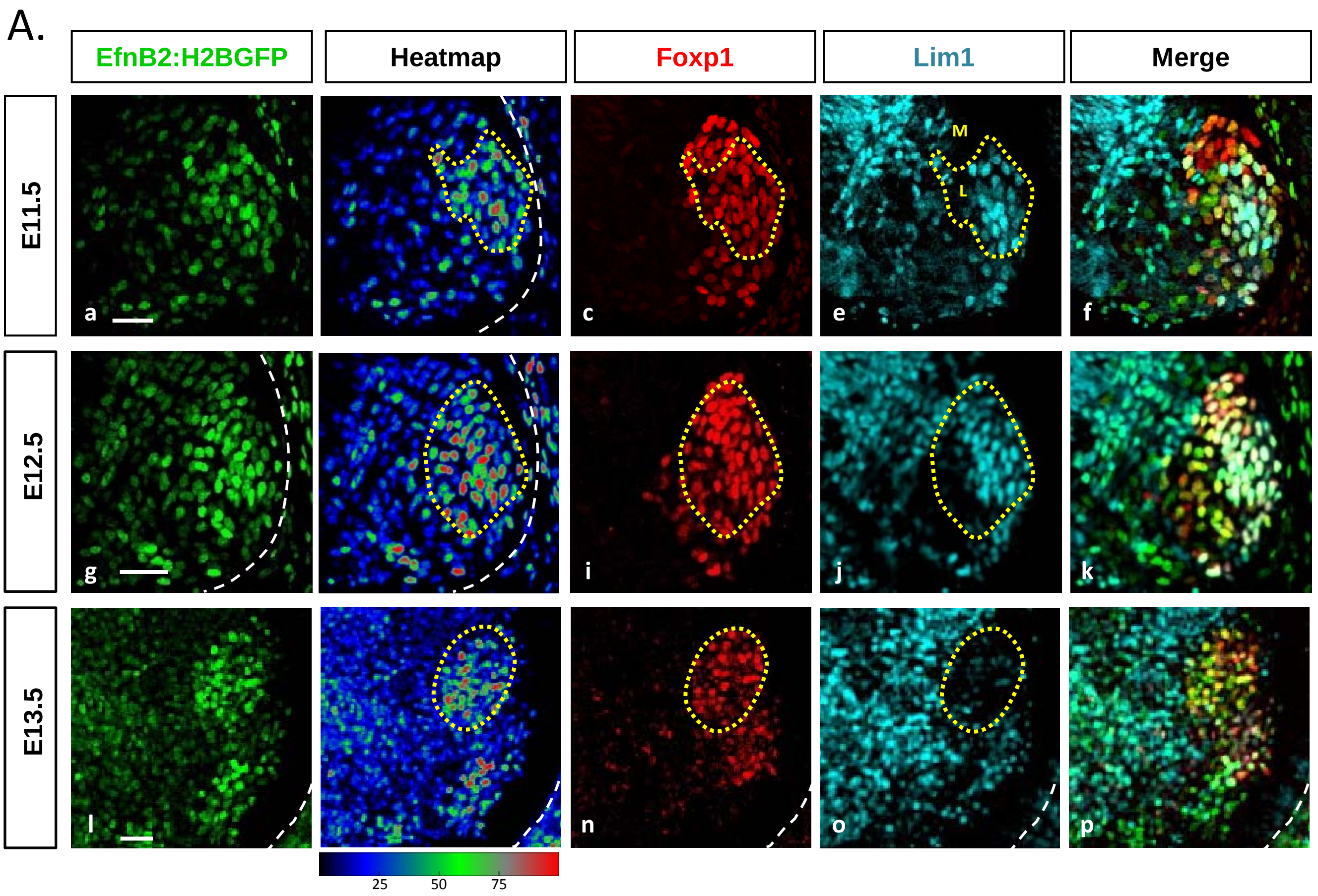

Sup Figure 1. Co-expression of ephrinB2 and Lim1.
Transverse vibratome sections of E11.5 (a-e), E12.5 (f-j), E13.5 (k-o) *EfnB2*$^{+/GFP}$ embryos were immunostained for Lim1 (c, h, m; red), Foxp1 (d, i, n; blue) showing LMCl and LMC neurons respectively. Epifluorescence from the GFP (a, f, k; green) is also represented on heat maps (b, g, l) to visualize cells expressing high levels of ephrinB2 (GFP^{high} in red). Dotted lines mark Foxp1^{+}/GFP^{high} cells. Scale bars: 50 µm.

Sup Figure 2, Luxey et al.

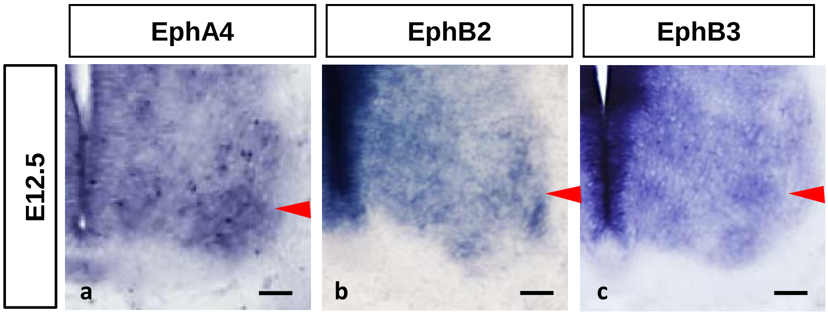

Sup Figure 2. Eph receptors are expressed in LMC MN.
Expression of *EphA4* (a), *EphB2* (b) and *EphB3* (c) was detected on transverse vibratome sections of E12.5 embryos by in situ hybridization.Scale bars: 50 µm.

Sup Figure 3, Luxey et al.

A.

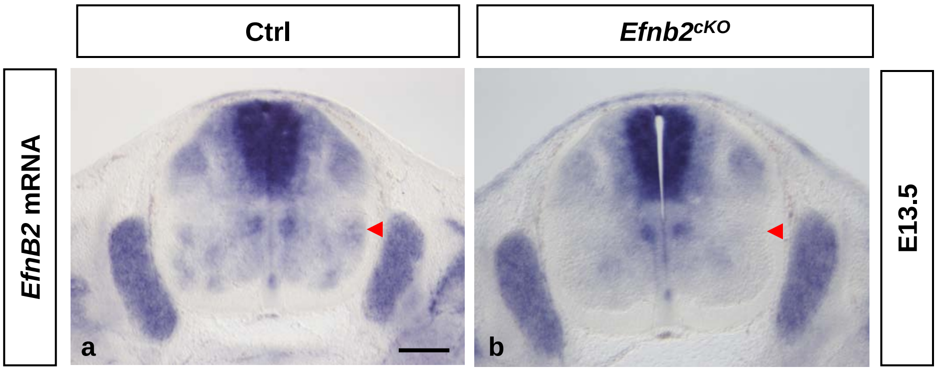

B.

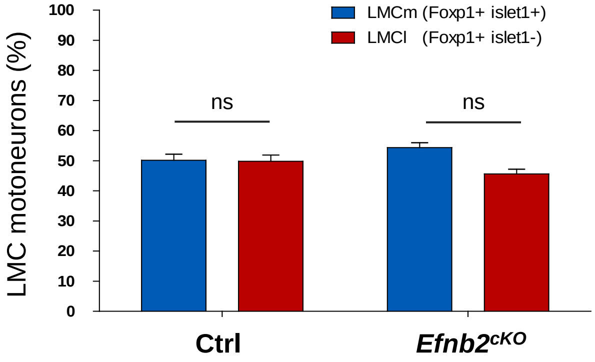

Sup Figure 3. Conditional excision of *Efnb2* in MN does not affect specification of LMC MN.

A. Transverse vibratome sections of control (*EfnB2*$^{+/GFP}$) and *Efnb2*^{cKO} (*EfnB2*$^{lox/GFP}$*; Olig2-Cre*) E13.5 embryos were processed for *Efnb2* in situ hybridization. Arrowheads indicate the position of the LMC. B. Transverse vibratome sections of control (*EfnB2*$^{+/GFP}$) and *Efnb2*^{cKO} (*EfnB2*$^{lox/GFP}$*; Olig2-Cre*) E13 embryos were immunostained for Foxp1 and Islet1 (see Figure 4). The graph shows the proportion of LMCm and LMCl MN in both genotypes. Error bars indicate s.e.m.; ns= non significant. Scale bars: 200 µm.
