# Supplementary material for: EphrinB2 sharpens lateral motor column division in the developing spinal cord
Source: Neural Dev. 2015 Oct 26;10:25. doi: 10.1186/s13064-015-0051-9 (PMC4624581; doi:10.1186/s13064-015-0051-9)
Supplement: Additional file 2: — Nearest Neighbor algorithm for R software. (DOC 28 kb) [file 13064_2015_51_MOESM2_ESM.doc]

**Supplementary methods**

***Nearest Neighbor algorithm for R software***

*neu=read.table("file.txt", h=T)* # load a new file

*head(neu)* # visualized file head

*dim(neu)* # visualized format

*attach(neu)* # fixing file without $

*X=c(xMN)* # c()=vector creation

*Y=c(yMN)*

*meandistanceNNA = function(x, y) {*

*nombre = length(x);*

*distVPP = rep(0,nombre);*

*for (indNr in 1:nombre) {*

*indAutres = (1:nombre) != indNr*

*indX = x[indNr];*

*indY = y[indNr];*

*dist = sqrt((x-indX)^2 + (y-indY)^2)*

*distVPP[indNr] = min(dist[indAutres]);*

*}*

*return(mean(distVPP))*

*}*

*test=subset(neu,neu$position=="1")*

*distTOT=NULL*

*distpos=NULL*

*for (i in 1:84)*

*{pos=neu[neu$position==i,]*

*distpos=distMoyenneVPP(pos$xMN,pos$yMN)*

*distTOT=c(distTOT,distpos)*

*distpos=NULL}*

*mean(distTOT)*

*write.table(distTOT, "meandistance.txt")*
